# An investigation for public acceptance of laparoendoscopic single-site surgery

**DOI:** 10.12669/pjms.293.3272

**Published:** 2013

**Authors:** Dong Wang, Hong-Wei Hou, Zhen-Ling Ji

**Affiliations:** 1 Dong Wang, MD, Southeast University Medical School, Nanjing, Jiangsu210009, China.; 2 Hong-Wei Hou, MD, Southeast University Medical School, Nanjing, Jiangsu210009, China.; 3 Zhen-Ling Ji, MD, PhD, Southeast University Medical School, Nanjing, Jiangsu210009, China.

**Keywords:** Laparoendoscopic single-site surgery (LESS), Investigation

## Abstract

***Objective*** ***: ***Laparoendoscopic single-site surgery (LESS) is the latest innovation in minimally invasive surgery with unconfirmed advantages. The public perception of LESS is the basis of carrying out the surgery.

***Methodology:*** Participants from the outpatient department were invited to rate, on a 5-point Likert scale, the important factors including scar, complications, cost, pain and hospital stay in choosing surgery. In addition, those who preferred LESS would continue to make their choices as the risks of LESS in above mentioned aspects rose.

***Results:*** About 85% of the questionnaires were included in the analysis. Complication was the most important factor with an average score of 4.77±0.43, followed by pain (3.84±0.96), scar (3.57±1.17), cost (3.41±0.87) and hospital stay (3.04±0.86). Of the 196 participants, 132 (67%) preferred LESS with younger age (35.3±10.64 versus 40.4 ±9.6, P=0.001). Better cosmesis was the only factor that made the participants choose LESS (3.78±1.11 versus 3.13±1.19, P<0.005). Almost 90% of the participants could accept the hypothesis (incision length of 3.5cm, cost up to 120%, pain up to 120%, hospital stay of 5 days), while only 50% of participants could accept the risk of complications of 6%.

***Conclusions:*** Complication is the most important factor that the public are concerned about in choosing surgery. LESS is preferred by young who care more concerned about the cosmesis, even with moderately elevated risks of extending incision and increasing hospital cost, postoperative pain and hospital stay.

## INTRODUCTION

Minimally invasive surgery has been decreasing the trauma of surgical operations and pushing surgical technique forward since Philip Mouret performed the first vedio-laparoscpic cholecystectomy in 1987.^1^ Thus, laparoscopic approaches have become currently the primary treatment for most of the surgical problems, including benign and malignant conditions. The advantages of the procedure compared to the open approach include decreased physical trauma, better cosmetic results, less postoperative pain and shorter recovery time. To further reduce operative trauma and improve cosmetic results, incisions of conventional laparoscopic approaches continue to decrease. Laparoendoscopic single-site surgery (LESS) is the latest innovation in minimally invasive surgery and benefited great development in recent years.^2^

LESS is performed through only one umbilical incision, using modifications of existing conventional laparoscopic technology, such as multiport trocar, bent or articulating instrumentations, and leaves an almost invisible scar, covered by the navel.^3^ Nowadays, LESS is getting widespread throughout the world, performed in general surgery, bariatric, urology and gynecology.^2^^,^^4^ However, LESS seems to be pushed forward mostly by the surgeons’ appetence for new developed instruments and thus its application reduces to experimentation with guinea pigs. Like conventional laparoscopic surgery (CLS), the introduction and dissemination of LESS should be driven by public acceptance and demand. Choice of surgical approach should be made by the patients, not by doctors or medical equipment manufacturers, especially at the moment that the benefit of LESS is not verified by abundant randomized controlled trials.^5^

Thus so far, several investigations have been done to assess the public perception for scarless surgeries, including LESS and natural orifice transluminal endoscopic surgery (NOTES).^6^^,^^7^Owing to the development of instruments and surgical technique, LESS is getting more clinical applications than NOTES.^8^ Unfortunately, there is no particular study investigating public perception for LESS and the possibly increasing unfavorable outcomes as a direct result of the surgery, such as complications, postoperative pain, cosmesis, hospital stay and cost. Thus, the aim of this study was to investigate the public perception of LESS and their tolerance of LESS for potential increasing risks, especially in the above-mentioned aspects, compared with CLS.

## METHODOLOGY

The survey was designed and approved by the Minimally Invasive Institute of Southeast University Medical School. The investigation was conducted in the outpatient department of the affiliated hospital. Two of the investigators (H.W. Hou and D. Wang) disseminated and recycled the questionnaires. The concepts of LESS and CLS were clearly described in the questionnaire and thus the investigators just sent out the questionnaires, without the need to explain anything about the investigation, avoiding prejudicial or biased comments on the surgical approaches. We planned to finish the survey in 10 days, with 24 questionnaires a day. In order to achieve a random sampling, the method of simple random sampling was used to identify the investigating days among the working days from May 2012 to June 2012. Then 12 patients were identified by table of random numbers from the first 100 outpatients (case no. 1-100) every investigating day in the outpatient department of general surgery. Additionally, one of the entourages of a patient was involved in the survey if they existed. If the corresponding patient of the case no. selected could not be found, the questionnaire was cancelled. Participants were excluded if they were less than 16 years old or they were cases of illiteracies. The survey was not conducted in the ward and the hospital staff was excluded in the study to avoid workplace bias. Participation in the study was voluntary, and no reward was offered for them. 

Suppose the participants got gallstones, and cholecystectomy was necessary in the near future. To avoid bias from the assigned surgical approaches in the questionnaire, the participants involved in the survey were not actual patients who were suffering from cholelithiasis. Brief information of CLS and LESS, including operation process, risks and advantages, was provided to the survey population. The questionnaire was present in Appendix. Elements in the questionnaire included two parts. One of them was investigating the factors with which participants were most concerned when choosing surgery, including abdominal wall scar, complications, cost, postoperative pain and hospital stay. Participants were asked to rate the importance of the above mentioned aspects on a 5-point Likert scale (1: not important, 5: very important). Then, participants were asked to make their choice whether they preferred LESS or CLS.

If preferred LESS, they would continue to ask whether they would prefer LESS when LESS was presented with increasing risks in cosmesis, complications, cost, postoperative pain and hospital stay. Besides, basic situation of the survey population was necessary in the questionnaire.

To calculate sample size, we assumed that 75% of the sample would prefer LESS, based on a recent study.^6^ With an alpha of 0.05 and one-sided deviation of 5% (Upper Limit), a sample size of 199 was required in the survey, calculated from the PASS software (version 08.0.16).Data extracted from the questionnaire were entered into a computerized spreadsheet for analysis. Categorical variables were reported as frequencies and percentages, and were analyzed by Chi-squared test. Continuous variables were reported as means and standard deviation, and were analyzed by independent sample t-test. P < 0.05 was considered clinically significant. The reported P values are two-tailed. SPSS statistical software (version 17.0) was used in the analysis.

## RESULTS

**Fig.1:**
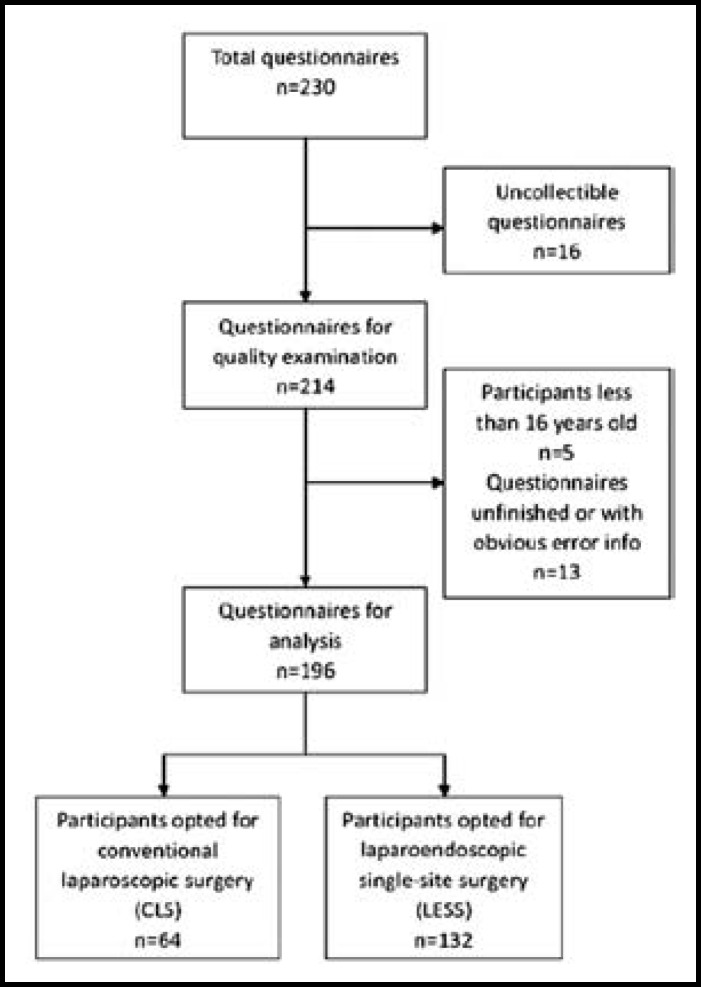
Participants inclusion process

***Demographics of Survey Population: ***From May 2012 to June 2012, 230 questionnaires were distributed and finally 196 (85%) were included in the analysis. The process of screening was shown in Fig.1, and the demographics of the survey population were presented in Table-I. Coincidentally, it was the same as the number of male and female. Of the survey population, 56% were between 30 and 50 years old, while only 14% of them were over 50 years old. 112 participants (64%) were college educated. The average BMI was 22 kg/m^2^, with a maximum of 29 kg/m^2^. And finally, 24 participants (12%) involved in the survey had history of previous surgery.

***LESS versus CL: factors related to the adoption: ***Of the 196 participants, 132 (67%) preferred LESS after they distinguished the differences between LESS and CLS. Age was the unique difference between the two groups. Participants preferring LESS had a composite life of 35.3±10.64, younger than those who preferred CLS being an average age of 40.4 ±9.6 (P=0.001). And, there were no significant differences between the two groups in gender, BMI, educational status and history of previous surgery as shown in Table-II.

**Fig.2:**
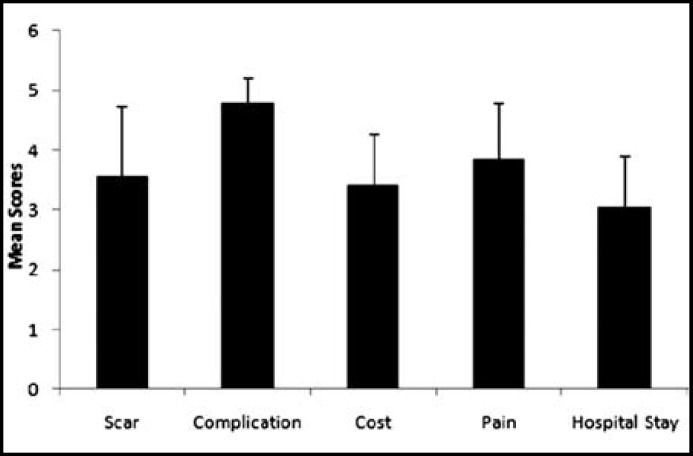
Factors concerned in choosing surgery

Complication was the most important factor that the participants would care about before they chose surgery, with an average score of 4.77±0.43, which was much higher than the other 4 factors. Postoperative pain was the second most important factor with a score of 3.84±0.96, and hospital stay got the lowest score of 3.04±0.86, as shown in Fig.2. For the participants preferring LESS, postoperative scar was the only difference compared with those preferring CLS (3.78±1.11 versus 3.13±1.19, P<0.05). And that attracted more attention from male than from their female counterparts (p=0.002 versus p=0.049), as shown in Table-II. There were no significant differences among the other 4 factors.

**Fig.3:**
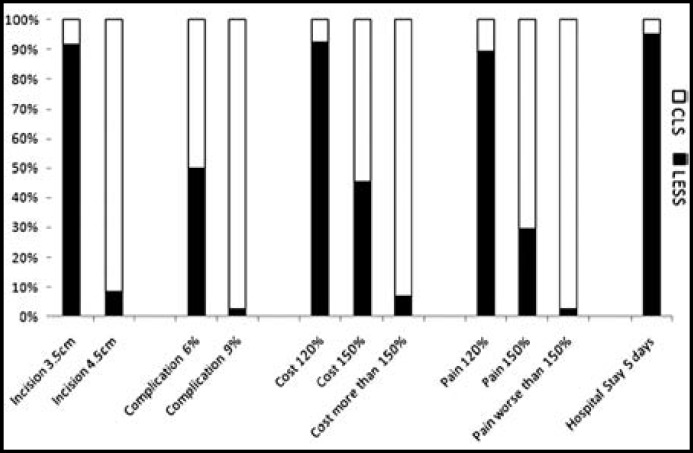
Acceptance of participants as the risks of LESS rose

***To what extent LESS could be accepted for? ***In the introduction of LESS for the survey population, LESS was described as an alternative of CLS, with the only difference being the location of the incisions on abdominal wall. And they had the similar advantages compared to traditional open surgery, such as decreased complications, shorter hospital stay, less hospital cost and improved postoperative pain. However, all of the operations must be done through the only one umbilical incision, and that must produce a lot of changes, with potential increasing risks of complications, pain, cost and hospital stay. The acceptance of participants as the risks of LESS rose was shown in Fig.3. Increased complications were the greatest obstacle to introduce LESS, which was accepted by only 50% of participants if the risk of complications increased to 6%. When the risk of complications increased to 9%, only 3 participants would like to choose LESS. 91.7% of the participants could accept a longer umbilical incision of 3.5cm, compared to CLS with a total incision length of 2.5cm. However, 11 participants (8.3%) preferred LESS even if the incision extended to 4.5cm. Participants had a similar attitude to the hospital cost and postoperative pain. Almost 90% of the participants would choose LESS to remove their gallbladder if the cost and pain increased up to 120%. When cost and pain increased to more than 150%, only 6.9% and 2.3% of the respective survey population would still prefer LESS. Surprisingly, 95.5% of participants would prefer LESS with a prolonged hospital stay of 5 days.

## DISCUSSION

Laparoscopic approach was introduced into surgical arena 25 years ago with a significantly increased risk of bile duct injury at that time, because of the blind pursuit of the new technology while ignoring the needs of the patients. The patients should get a comprehensive understanding of the disease and surgery before signing the informed consent. The choice of the surgical approach should be made by the patients according to their own situations, not by the surgeons. As a new surgical approach, LESS should be carefully assessed by the surgeons and patients, to prevent the unexpected damage from happening again.^9^ Thus, to survey the needs of patients was necessary and important at the moment that the theoretical advantages of LESS were not confirmed by abundant randomized controlled trials, such as better cosmesis, improved postoperative pain, decreased complications, less cost and shorter hospital stay. In this study, we investigated for the first time, the concerns of participants in choosing surgery and the tolerance of the potential increasing risks of LESS. 

Consistent with the recent studies,^6^^,^^10^^,^^11^ complication was the most important factor which the participants were concerned with in choosing surgery. Thus, preventing the incidence of complications from increasing was the most important task for the prosperity of LESS, which was the reason why LESS got more clinical applications rather than NOTES. And it corresponded to the result that the participants preferring LESS decreased apparently if the complications of LESS increased from 3% to 6%. Most of the participants preferring LESS were not concerned with the hospital stay, even if it prolonged to 5 days. Maybe it was because of the social insurance system and social habit, which was similar to the situation in the East Asian countries. More than 90% of the participants preferring LESS would still prefer LESS even if the hospital cost increased up to 120%. That was important information for surgeons, because a lot of new developed instruments with relatively high price could be introduced into LESS, such as multichannel platform and articulating instruments, which could ameliorate the operation of LESS.

**Table-I:** Demographics of survey population

*Demographics*	*Participants*
Gender (male/female)	98/98
Age (means, std.deviation)	36.97,10.56
≤30 years old (frequency)	60
30-50 years old (frequency)	109
≥50 years old (frequency)	27
Educational Status (basic /higher education)	84/112
BMI (means,std.deviation)	21.96,2.86
History of Previous Surgery (yes/no)	24/172

**Table-II:** LESS versus CLS in demographics and important factors

	*LESS*	*CLS*	*Sig. P †*
*Gender (male/female)*	62/70	36/28	0.223
Age (*)	35.30(10.64)	40.41(9.60)	0.001
Educational Status (basic /higher education)	55/77	29/35	0.629
BMI (*)	22.09(2.96)	21.68(2.65)	0.345
Male	23.40(2.80)	22.75(2.69)	0.260
Female	20.93(2.61)	20.31(1.91)	0.252
History of Previous Surgery (yes/no)	19/113	5/59	0.187
Postoperative Scar (*)	3.78(1.11)	3.13(1.19)	0.000
Male	3.23(1.03)	2.58(0.87)	0.002
Female	4.27(0.93)	3.82(1.19)	0.049
Complications (*)	4.76(0.45)	4.80(0.41)	0.553
Surgical Cost (*)	3.47(0.94)	3.28(0.72)	0.158
Postoperative Pain (*)	3.79(0.98)	3.95(0.93)	0.263
Male	3.23(0.90)	3.61(0.99)	0.051
Female	4.29(0.76)	4.40(0.63)	0.512
Hospital Stay (*)	3.00(0.90)	3.11(0.78)	0.406

*indicates means (std.deviation). †was calculated in 2-tailed. LESS: Laparoendoscopic single-site surgery. CLS: Conventional laparoscopic surgery

**Legends:**

**LESS: **Laparoendoscopic single-site surgery.

**CLS:** Conventional laparoscopic surgery.

 Improved postoperative pain was believed to be one of the potential advantages for LESS, compared with CLS. However, there were some controversies in the recently published studies. In the study of Lee,^12^ postoperative pain scores and analgesic requirements were similar for both groups of LESS and CLS. And in another RCT article,^13^ which included the largest number of patients for cholecysectomy, pain scores were lower for CLS despite equal analgesia use. But in another RCT research,^14^ significantly lower pain scores were observed in the LESS group versus the CLS group after the first 12h for abdominal pain and after the first 6h for shoulder pain. However, it was encouraging that postoperative pain management should be treated in a moderate attitude. Because almost 90% of participants preferring LESS would like to choose LESS when the pain increased to 120%, and 30% of the participants would still prefer LESS even if the pain grew up to 150%.

Better cosmetic result was verified in some RCTs,^15^^-^^17^ which was also confirmed in our data. Most of the participants believed that the unique umbilical incision had better cosmetic results than 3-4 separate abdominal wall scares. And 91.7% of the participants preferring LESS would prefer LESS even if the umbilical incision prolonged to 3.5cm. However, LESS was not recommended for those surgeries in which the big specimens needed to be removed from the abdomen as a whole, such as malignant tumor more than 4.5cm. Because less than 10% of the participants would choose LESS if the umbilical incisional length was more than 4.5cm. Pursuit of cosmesis was consistent with the difference of age between the two groups of LESS and CLS, and the aptness was not influenced by the sex.

There were several limitations in our study. First, the participants were all recruited from the outpatient department, and it would bring in some bias of location. The second limitation was the distribution of age, only 14% of the participants being over 50 years old. Another possible weakness was the relatively small number of participants.

## CONCLUSION

 Complication is the most important factor that the public are concerned about in choosing surgery. LESS is preferred by young who care more about the cosmesis, even with moderately elevated risks of extending incision and increasing hospital cost, postoperative pain and hospital stay.

## Authors Contribution

*Dong Wang* and *Hong-Wei Hou* performed the survey, and *Zhen-Ling Ji* designed the study and managed the manuscript.
